# Polarity effects, resistance, and probiotic enhancement of intoxication of *Salmonella enterica fraB* mutants in murine models

**DOI:** 10.1371/journal.pone.0339602

**Published:** 2025-12-31

**Authors:** Anice Sabag-Daigle, Erin F. Boulanger, Maryam Baniasad, Yongseok Kim, Madalyn Moore, Bailyn Hogue, Andrew Schwieters, Venkat Gopalan, Vicki Wysocki, Brian M. M. Ahmer

**Affiliations:** 1 Department of Microbial Infection and Immunity, The Ohio State University, Columbus, Ohio, United States of America; 2 Department of Chemistry and Biochemistry, The Ohio State University, Columbus, Ohio, United States of America; 3 Department of Microbiology, The Ohio State University, Columbus, Ohio, United States of America; NMIMS Deemed to be University - Mumbai Campus: NMIMS, INDIA

## Abstract

FraB is a deglycase in a metabolic pathway that allows *Salmonella* to utilize fructose-asparagine (F-Asn). Some *fraB* mutants are sensitive to F-Asn due to the accumulation of 6-phosphofructose-aspartate (6-P-F-Asp), a toxic intermediate in this pathway. We determined that different alleles of *fraB* cause different amounts of 6-P-F-Asp-mediated toxicity due to effects on the expression of the downstream gene, *fraD*, a kinase. Mutations in *fraD* or *fraA* (a transporter) cause resistance to F-Asn intoxication, and these mutations occur during infection. To better mimic the effect of a hypothetical FraB inhibitor in mouse models, we characterized a non-polar mutant encoding a catalytically inactive FraB (FraB E214A). We also compared a typical mouse chow and a high-fat chow and found that the latter decreases the variation in colonization typically observed during infection of CBA/J mice with *Salmonella*. Because the high-fat chow lacks F-Asn, the *fraB* E214A mutant was not attenuated in mice fed this diet unless F-Asn was supplemented. F-Asn supplementation resulted in a 100-fold reduction of colony forming units (CFU) recovered from feces compared to wild-type. Co-infection of *Salmonella* with a *Salmonella* “probiotic” strain that is neither virulent nor capable of consuming F-Asn (a SPI1 SPI2 *fraR-BDAE ansB* mutant) led to a dramatic 10,000-fold reduction in CFU and a 1000-fold reduction in lipocalin-2, a proxy marker of inflammation. This probiotic strain presumably competes for nutrients other than F-Asn, driving the *fraB* mutant to consume a higher proportion of F-Asn and greater 6-P-F-Asp intoxication. Thus, a putative inhibitor of FraB, when administered with F-Asn and a probiotic, may provide a new therapeutic strategy for treating *Salmonella* gastroenteritis.

## Introduction

*Salmonella enterica* is one of the top three causes of food-borne infections in the United States and is a leading cause of hospitalization and deaths [1]. Globally, diarrheal disease is a major cause of morbidity and mortality, especially among children, of which non-typhoidal *Salmonella* infections from contaminated food and water are a major contributing factor [2,3]. *Salmonella enterica* subspecies *enterica* includes many serovars that cause acute gastroenteritis, characterized by fever and diarrhea. Here, we study serovar Typhimurium, which we will refer to as *Salmonella*. Disease pathology is marked by extensive intestinal inflammation mediated by the two *Salmonella* pathogenicity islands that encode type three secretion systems, SPI1 and SPI2 [4,5]. Inflammation disrupts the microbiota, eliminating competitors for nutrients and providing alternative electron acceptors for respiration [4,6–13]. Currently, broad-spectrum antibiotics are used to treat only high risk but not uncomplicated cases, largely because these drugs disrupt the normal microbiota and lead to higher susceptibility to the pathogen and increased shedding of *Salmonella* over time [14–18]. Resistance of microbial pathogens to existing antibiotics is a growing problem that necessitates novel therapeutics. Both the CDC and the WHO have released reports stating that new drugs are needed for the non-typhoidal *Salmonella* serovars due to increasing fluoroquinolone resistance [19,20].

**Fig 1:**
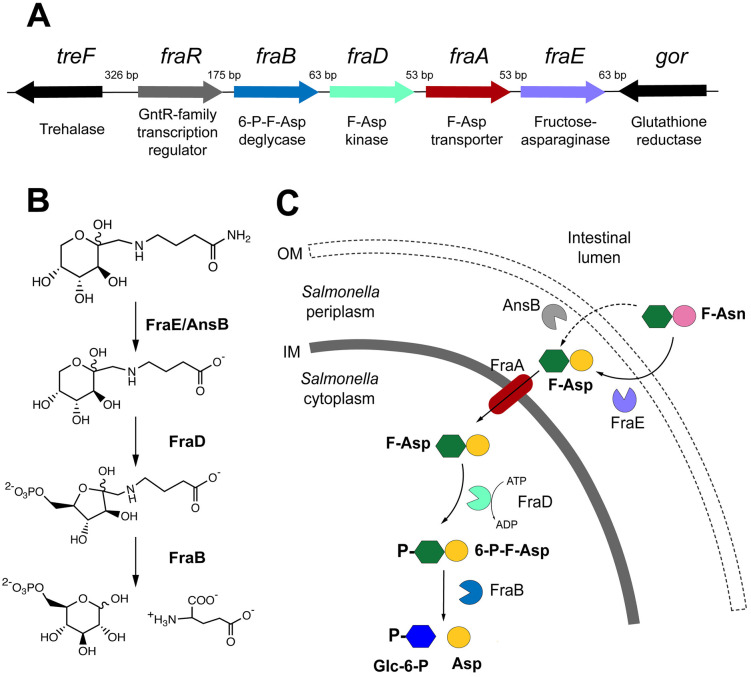
Fructose-asparagine metabolism in *Salmonella enterica.* **A)** *Salmonella* genes involved in the metabolism of fructose-asparagine. The sizes of intergenic regions are indicated above the operon depiction. **B** and **C)** Proposed pathway for metabolism of F-Asn, including locations of the transporter and catabolic enzymes.

A gainful strategy for identifying new drug targets is to use one of several parallel screening strategies to identify genes that contribute to bacterial fitness during infection [21–23]. An early example of these strategies, called Transposon Site Hybridization (TraSH), revealed that mutations in the previously uncharacterized *fra* locus of *Salmonella* are highly attenuated in several mouse models [24]. The *fra* locus is a horizontal acquisition encoding five genes that encode a transcription factor, a transporter, and three enzymes, all involved in the uptake and catabolism of fructose-asparagine (F-Asn) (Fig 1) [25–27]. F-Asn is an Amadori product formed by the non-enzymatic condensation of asparagine and glucose [24,28,29]. F-Asn enters the periplasm of *Salmonella* where FraE, a fructose-asparaginase, releases ammonia from F-Asn to make fructose-aspartate (F-Asp) [26] (Fig 1B, 1C). AnsB, a periplasmic asparaginase, can also deamidate fructose-asparagine and partially complement a *fraE* mutant [26]. FraA, a DcuA-family transporter, transports F-Asp into the cytoplasm where it is phosphorylated by FraD forming 6-phosphofructose-aspartate (6-P-F-Asp) [25]. FraB, a deglycase, converts 6-P-F-Asp to glucose-6-phosphate (Glc-6-P) and aspartate [27]. A *fraB80*::kan mutant accumulates 6-P-F-Asp, displays reduced growth, and has greatly reduced fitness during infection of mice via the oral route [24,30]. This growth inhibition is bacteriostatic as growth resumes upon removal of F-Asn. However, within the murine gastrointestinal tract, F-Asn is bactericidal to the *fraB80*::kan mutant as the number of bacteria decline over time [30]. The mechanism by which F-Asn inhibits the *fraB* mutant of *Salmonella* is unknown. Throughout this report, we refer to the inhibition of the *fraB* mutant by F-Asn as intoxication.

F-Asn can be found in a number of human foods and is especially prevalent in dessicated foods such as dried apricots [31]. The concentrations present in these foods are physiologically relevant as the IC_50_ for F-Asn on a *fraB* mutant of *Salmonella* is between 10 and 30 μM ( [30] and this report) while the concentration of F-Asn present in mouse chow is approximately 10-fold higher than this, and the concentration in some human foods is 800-fold higher (dried apricots) [31]. We also determined that the genes of the *fra* locus likely originated in the Clostridia [32]. Among Gram-negative bacteria, the *fra* genes are found only within *Salmonella*, and a few close relatives in the *Klebsiella* and *Citrobacter* genera [32]. Mutants lacking *fraB*, *fraD*, or *fraA* are unable to utilize F-Asn as sole carbon source. However, mutants lacking *fraD* or *fraA* can grow on other nutrients in the presence of F-Asn (i.e., F-Asn negative) while a *fraB* mutant cannot (i.e., F-Asn sensitive). This finding suggested that the *fraB* mutant was somehow intoxicated by F-Asn, and we determined that 6-P-F-Asp (the FraB substrate) indeed accumulates in the *fraB* mutant and is likely to be the intoxicating compound [30].

Upon ingestion, F-Asn is consumed by specific members of the normal intestinal microbiota, primarily members of the class Clostridia [7,32]. *Salmonella*-mediated inflammation eliminates these organisms, allowing *Salmonella* access to the nutrient [7]. *Salmonella* typically fails to induce inflammation in mice colonized with a healthy microbiota and thus fails to gain access to F-Asn. However, treatment of laboratory mice (e.g., Swiss-Webster) with streptomycin dramatically reduces the population of Clostridia and allows *Salmonella* to quickly inflame the intestinal tract and gain access to F-Asn [6,7,24,30,33]. The use of antibiotics to disrupt the microbiota is not always essential [34–36]; for example, CBA/J mice are unusual in that they allow long term intestinal colonization by *Salmonella*, which eventually leads to inflammation (at 8–10 days post-infection) in roughly 30% of the mice. Here, we report that switching CBA/J mice to a high-fat chow allows all the mice to become inflamed by *Salmonella*. Additionally, because the high-fat chow does not include F-Asn, we show that F-Asn supplementation is necessary for a *fraB* mutant to be attenuated in mice. We determine that different mutant alleles of *fraB* have very different phenotypes with regard to intoxication by F-Asn *in vitro* and in mouse models. We also demonstrate that coinfection of a *Salmonella fraB* mutant with a probiotic strain that cannot utilize F-Asn enhances the mutant’s attenuation and greatly reduces inflammation in the gut. These results collectively suggest that presumptive inhibitors of FraB, when administered with F-Asn and a probiotic, would represent a new therapeutic strategy to treat *Salmonella*-mediated gastroenteritis.

## Materials and methods

### Bacterial culture media

All bacterial cultures were maintained in LB broth (Fisher BioReagents) or on agar plates with 1.5% (w/v) agar (Fisher BioReagents). Strains used in this study are listed in Table 1. Antibiotics were added to media for plasmid maintenance or marker selection at the following concentrations: kanamycin (kan, 50 μg/ml), chloramphenicol (cam, 30 μg/ml), ampicillin (amp, 100 μg/ml). Ten mM EGTA was used to stop P22 phage infection. Screening for pseudolysogens after P22 transductions entailed the use of Evans Blue-Uranine (EBU) plates consisting of (per L): 10 g tryptone, 5 g yeast extract, 5 g NaCl, 1.5 g glucose, 1.5% (w/v) agar. After autoclaving, the medium was supplemented with 40 mL of 12.5% (w/v) K_2_HPO_4_, 1.25 mL 1% (v/v) Evans Blue solution, and 2.5 mL 1% (w/v) Uranine [37].

**Table 1:** Strains and plasmids.

Strain	Genotype	Reference
14028	Wild-type *Salmonella enterica* subspecies *enterica* serovar Typhimurium	American Type Culture Collection
*E. coli* 1917 (Nissle)	*E. coli* Nissle serotype O6:K5:H1	[38]
ASD5900	14028 Δ*fraB1*::cam	P22 transduction of Δ*fraB1*::cam from MA5900 into 14028.
ASD4353	14028 Δ*fraB1*	Antibiotic cassette in ASD5900 was removed using Flp recombinase encoded by pCP20.
ASD4324	14028 Δ*fraB53*::cam	lambda red mutation of *fraB* made using PCR primers BA3407 and BA3408 with pCLF3 as template and transduced into a clean 14028 background using phage P22.
ASD4325	14028 Δ*fraB53*	Antibiotic cassette in ASD4324 was removed using Flp recombinase encoded by pCP20.
ASD1312	14028 *fraB* E214A	[27]
ASD4355	14028 *fraB* E214A IG(*pagC-STM14_1502*)::cam	Transduction of IG(*pagC-STM14_1502*)::cam from JLD1214 into ASD1312 using phage P22.
HMB184	14028 *fraD4*::kan	[30]
HMB205	14028 Δ(*fraR-BDAE*)*80*::kan	[30]
HMB206	14028 Δ*fraB80*::kan	[30]
HMB216	14028 Δ*fraB80*	Antibiotic cassette in HMB206 was removed using Flp recombinase encoded by pCP20.
HMB247	14028 *fraA4*::kan	[30]
JLD1214	14028 IG(*pagC-STM14_1502*)::cam	[24]
MA5900	14028 Δ(*avrA-invH*)*1ssaK*::kan *fraB1*::cam	[24]
ASD200	14028 Δ(*avrA-invH*)*1* Δ(*ssrB-ssaU*)*1*	[39]
ASD201	14028 Δ(*avrA-invH*)*1* Δ(*ssrB-ssaU*)*1 Δ(fraR-fraBDAE)4::Kan*	[39]
ASD202	14028 Δ(*avrA-invH*)*1* Δ(*ssrB-ssaU*)*1 Δ(fraR-fraBDAE)4*	Antibiotic cassette in ASD201 was removed using Flp recombinase encoded by pCP20.
ASD203	14028 Δ(*avrA-invH*)*1* Δ(*ssrB-ssaU*)*1 Δ(fraR-fraBDAE)4 ΔansB80*::kan	Transduction of ansB80::kan from HMB107 into ASD202 using phage P22.
ASD204	14028 Δ(*avrA-invH*)*1* Δ(*ssrB-ssaU*)*1 fraB80*::kan	Transduction of *fraB80*::kan from HMB206 into ASD200 using phage P22.
ASD4320	14028 *ttrA1*::cam	Transduction of *ttrA1*::cam from MA4320 into 14028 using phage P22
MA4320	IR715 *ttrA1*::cam	Transduction of *ttrA1*::cam from the BEI resources collection [40] into IR715 using phage P22
HMB249	14028 *fraB80*::kan *ttrA1*::cam	Transduction of *ttrA1*::cam from MA4320 into HMB206 using phage P22
IR715	14028 nal^r^	[41]
TH3923	pJS28(Ap P22-9+)/F’114ts Lac + zzf-20::Tn10[tetA::mudP](TcS) zzf-3823::Tn10dTc[del-25]/leuA414 hsdSB Fels2-	[42]
**Plasmids**		
pCLF3	FRT-*cam*-FRT *oriR6K* (amp^r^)	[40]
pKD4	FRT-*kan*-FRT *oriR6K* (amp^r^)	[43]
pKD3	FRT-*cam*-FRT *oriR6K* (amp^r^)	[43]
pCP20	cI857 λPR *flp* pSC101 *oriTS* (amp^r^ cam^r^)	[44]

### Growth assays

For growth studies with minimal medium, we used M9 minimal medium supplemented with 19 mM ammonium chloride as previously described [30]. F-Asn was synthesized as previously described [29]. Growth of bacteria was measured in a 96-well clear, flat bottom plate with M9 minimal medium supplemented with the specified carbon sources at the concentrations stated and inoculated 1:100 with an overnight culture that had been washed twice in sterile water [30]. Growth was measured with hourly absorbance measurements (600 nm) at 37°C for 15 h in a SpectraMax i3X (Molecular Devices) microplate reader and the SoftMax Pro 6.1 software.

### Minimum inhibitory concentration

The MIC for each strain was calculated from a growth assay after 15 h incubation at 37°C using three biological replicates with three technical replicates each (nine total data points). IC_50_ and IC_90_ values were calculated with Prism (GraphPad).

### Mutant construction

Lambda Red mutagenesis was used to construct in-frame deletion mutants of targeted genes in *Salmonella* as previously described using primers listed in Table 2 [43]. Some mutations were constructed using pKD3 or PKD4 as PCR templates, while others were constructed using pCLF3 or pCLF4 as template [40,43]. Others were taken from the BEI resources collection [40] which were constructed similarly using pCLF3 or pCLF4 as templates, and then through phage P22 transduction moved into 14028, our laboratory’s wild-type strain (see below). Antibiotic cassettes were removed by electroporating with pCP20 which encodes FLP recombinase, as previously described [43].

**Table 2:** Oligonucleotides used in this study.

Name	Sequence (5’ to 3’)	Description of use
BA2745	CGAAGCGGCTGAAAGCCTGT	*rpoB* qRT-PCR forward primer
BA2746	TTCGATTTCGTCGCGCAGCA	*rpoB* qRT-PCR reverse primer
BA3405	ACCTGGAATCGGAACTGGAG	*fraD* qRT-PCR forward primer
BA3406	TTTCATCTGGCGATAACCCG	*fraD* qRT-PCR reverse primer
BA3407	GAACCAGAGGAAAGCATGATGGGTATGAAAGA GACAGTTAGCAATCATATGAATATCCTCCTTAG	Forward primer for *fraB* lambda red mutagenesis
BA3408	CCTGATGTAATTAATATTCCACTTTCCACATATA GCGGCGGTGTAGGCTGGAGCTGCTTC	reverse primer for *fraB* lambda red mutagenesis

### P22 transductions

Alleles were transduced from one strain to another using phage P22HT*int*. To accomplish this goal, phage P22HT*int* was propagated on the donor strain and the resulting phage were used to infect the recipient strain. Transductants were selected on LB supplemented with the appropriate antibiotic and 10 mM EGTA. Colonies were streaked to isolation twice on this same medium. Transductants were then tested for pseudolysogeny and full length LPS by streaking against P22HT*int* on EBU plates [37].

### *fraD* qRT-PCR

Bacterial strains were grown in LB broth using a roller drum at 37°C overnight. The cultures were then washed twice and resuspended in sterile water. The washed cells (500 μL) were used to inoculate 50 mL of M9 minimal medium supplemented with 5 mM glucose resulting in a 1:100 dilution of cells. These cultures were allowed to grow for 4 h, F-Asn was then added to 1 mM, and growth was continued for 30 min before withdrawing 15-mL aliquots for intracellular 6-P-F-Asp measurements and qRT-PCR. The cells were harvested by centrifuging at 5,000 x g for 10 min at 4°C. Pellets were stored at −20°C. From the qRT-PCR aliquots, RNA was extracted using the Purelink RNA Miniprep kit (Invitrogen). Briefly, pellets were thawed on ice and resuspended in freshly made lysis buffer and lysed cells run through the column per the manufacturer’s protocol. After the RNA was eluted from the column in 50 μL RNase-free water, residual DNA contaminants were removed by treatment with one-tenth volume of 10x DNase buffer and 2 U of DNase (Turbo DNA-*free* kit; Invitrogen) for 30 min at 37°C. At the end of this incubation, 5 μL of DNase inactivation reagent was added to the reaction, incubated for 5 min at room temperature (~23°C) with periodic mixing, and centrifuged at 19,000 x g for 1 min. The supernatant was removed and placed in an RNase-free microcentrifuge tube and stored at −20°C. For reverse transcription (RT), 100 ng of total RNA was used for cDNA synthesis using Superscript VILO (Invitrogen) per the manufacturer’s protocol. For quantitation of *fraD* expression, 1 μL of cDNA was used in a qPCR reaction using iTaq SYBR Green Supermix (BioRad) with either 300 μM of primers BA3405 and BA3406 (*fraD)* or BA2745 and BA2746 (*rpoB*) in the CFX96 Touch Real-time PCR machine (BioRad). Amplification efficiencies for each primer pair were calculated using a 10-fold dilution series standard curve of pooled cDNA from all samples from one of the biological replicates (*fraD* BA3405 and BA3506 was 91.48%; *rpoB* BA2745 and BA2746 was 90.42%; see Table 2). The C_t_ values were analyzed using the Pfaffl method where the *rpoB* control was used as the internal calibrator and the wild-type *fraD* expression is the reference condition and *fraD* expression in all mutant backgrounds is relative to the wild-type [45].

### Measurement of 6-P-F-Asp concentration in *Salmonella* cells

To measure intracellular 6-P-F-Asp, 15-mL samples of cells were collected, pelleted (see qRT-PCR section above), and resuspended in 300 μL of LC grade water. Biological replicates were collected on three separate occasions. Each replicate was split into three aliquots of 100 μL. Two aliquots were used as analytical replicates for each analysis. 6-P-F-Asp was extracted from each 100 μL aliquot (equivalent to 5 mL of cells), by adding 250 μL of chilled LC grade water and 500 μL of chilled methanol (Fisher Optima LC/MS grade, Fisher Scientific) with 20 nmol [^13^C]-F-Asn added as internal standard. The cell suspension was vortexed for 2 min to facilitate pellet disruption and metabolite extraction. Samples were subjected to ten cycles of ultrasonication (30 s on, 30 s off cycles) using a Bioruptor® Pico (Diagenode). Following cell lysis, samples were centrifuged at 16,000 × g for 15 min at 4°C and the supernatants were transferred to new tubes and dried under vacuum (SpeedVac Concentrator, Thermo Scientific). Before mass spectrometry analysis, these dried pellets were resuspended in 50 μL water:acetonitrile (98:2) with 0.1% (v/v) formic acid (LC-MS grade, Thermo Scientific) and filtered using a 0.2-µm PTFE filter (Thermo Scientific). The supernatant of the flow-through fraction was then injected for liquid chromatographic-mass spectrometric analysis.

To construct a standard curve, different amounts of standard 6-P-F-Asp (0, 20, 80, 160, 200, 400 or 600 nmol) were spiked into the extraction solvent and were prepared as described above. Ten μL of each sample (equivalent to 1 mL of cells) was introduced into an Ultimate 3000 Ultra Performance LC (Thermo Scientific) system with an HSS T3, C18 column (Waters, 2.1 μm × 100 mm, 1.8 µm) and coupled into a triple quadrupole mass spectrometer (Thermo Quantiva TQ-S). Mobile phases were A [0.1% (v/v) formic acid (LC-MS grade, Thermo Scientific) in water] and B [0.1% (v/v) formic acid in acetonitrile]. The separation started with 2% B for 3 min at a flow rate of 100 μL/min and was then followed by a gradient: 3–4 min, 2–10% B; 4–7 min, 10–98% B; 7–7.5 min, 98% B; 7.5–7.6 min, 98–2% B; 7.6–10 min, 2% B. The mass spectrometer was operated in positive ion electrospray ionization mode (ESI+) with a capillary voltage at 4 kV, source temperature 100°C, desolvation temperature 350°C, sheath gas flow 12 L/min and auxiliary gas flow 13 L/min. The gas flow rate for the collision cell was 0.15 mL/min. A multiple reaction monitoring (MRM) mode was used. While the m/z 376 → 242 transition of 6-P-F-Asp with collision energy 15eV was selected for quantitation, m/z 301 → 283 of [^13^C]-F-Asn with collision energy 13eV was used for normalization. Skyline (v20.2, MacCoss Lab, Department of Genome Sciences, University of Washington, Seattle, WA, USA) was used for calculating the peak area of transition.

### Measurement of F-Asn in mouse fecal samples

For sample preparation, 1 mL of the extraction solvent (water/acetonitrile, 80/20) with [^13^C]-F-Asn was added to the fecal sample for F-Asn extraction. The fecal slurry was lysed by the bath sonication with Bioruptor® (Diagenode, Belgium) followed by centrifugation for protein precipitation. The supernatant was dried and reconstituted in water-acetonitrile-formic acid (98/2/0.1, v/v) before LC-MS/MS analysis. For the standard-curve analysis, different amounts of F-Asn standard compound (5.15, 12.9, 25.8, 51.5, 128.8, 257.7, 515.5, 1288.6 nmol) were spiked into the extraction solvent and were prepared as described above. F-Asn and [^13^C]-F-Asn were separated using Dionex UltimateTM 3000 RSLC liquid chromatography system (Thermo Scientific, USA) with a C18 column (Kinetex® 1.7 µm C18 100 Å, LC Column 100 x 2.1 mm, Phenomenex Inc, USA). Mobile phases were A [0.1% (v/v) formic acid (LC-MS grade, Thermo Scientific) in water] and B [0.1% (v/v) formic acid in acetonitrile]. The separation started with 2% B for 3 min at a flow rate of 100 μL/min and was then followed by a gradient: 3–7 min, 2% to 98% B; 7–7.5 min, 98% B; 7.5–10 min, 2% B. Thermo Quantiva triple quadrupole mass spectrometer (Thermo Scientific, USA) was used in a positive-ion mode. Transition conditions, including retention time, precursor ion, product ion, and collision energy, are described in Table 3. Skyline (v20.2, MacCoss Lab, Department of Genome Sciences, University of Washington, Seattle, WA, USA) was used for calculating the peak area of transition.

**Table 3:** Transition conditions for F-ASN and ^13^C_6_-F-ASN for LC-MS analysis.

Compound	Retention time (min)	Q1 (m/z)	Q2 (V)	Q3 (m/z)
Fructose-asparagine	1.95	295	17	211
		295	10	259
		295	11	277
^13^C_6_-Fructose-asparagine	1.95	301	17	216
		301	10	265
		301	11	283

### Measurement of F-Asn in mouse chow using mass spectrometry

For sample preparation, the mouse chow was lyophilized and approximately 10 mg was aliquoted into a 1.5 mL microcentrifuge tube and weighed. Five hundred μL of methanol and 500 uL of water was added as an extraction solvent and the sample was spiked with 1 μL of 2.58 mM [13C]-F-Asn. The sample was vortexed and centrifuged at 14,800 x g for 1 hour. The supernatant was transferred to a clean 1.5 mL tube and allowed to dry. For the standard curve analysis, different amounts of F-Asn standard (ranging from 0.000001 to 1.00 nmol) were spiked into the extraction solvent. After drying, the samples were reconstituted in 50 μL of 80/20 acetonitrile/water with 0.1% (v/v) formic acid (LC − MS grade, Thermo Scientific) and injected for mass spectrometry analysis. F-Asn and [13C]-F-Asn were separated using a Dionex UltimateTM 3000 RSLC liquid chromatography system (Thermo Scientific, USA) with an Acquity UPLC BEH Amide column (1.7 µm, 130 Å, 2.1 mm x 150 mm, Waters Corporation, USA). Mobile phases were A [0.1% (v/v) formic acid (LC-MS grade, Thermo Scientific) in 90/10 water/acetonitrile (v/v)] and B [0.1% (v/v) formic acid in acetonitrile]. The sample injection volume was 1 μL. Gradient elution was applied at a flow rate of 300 μL/min. The gradient began with 80% B for 6 minutes followed by 6 − 20 min, 80 − 50% B; 20 − 26 min, 50% B; 26 − 28 min, 50 − 80% B; 28 − 35 min, 80% B. A Thermo Quantiva triple quadrupole mass spectrometer (Thermo Scientific, USA) was used in a positive-ion mode. Transition conditions, including retention time, precursor ion, product ion, and collision energy, are described in Table 3. Skyline (v 22 MacCoss Lab, Department of Genome Sciences, University of Washington, Seattle, WA, United States) was used for calculating the peak area of transitions. The detection limit of F-Asn was determined to be 50 nM.

### Mouse experiments

For competition experiments, a 1:1 mixture of two different bacterial strains were inoculated intragastrically (i.g.) and then recovered from ceca to determine the competitive index (CI) of the two strains. Streptomycin-treated mice were administered 20 mg of streptomycin 24 h prior to inoculation with *Salmonella* strains [46]. Streptomycin-treated mice were inoculated with 10^7^ total CFU of *Salmonella* while mice that were not treated with streptomycin were inoculated with 10^9^ total CFU. CI was determined by plating on media selective for WT (LB cam) and mutant (LB kan). For the competitions with the *fraB* E214A mutant, which is not marked, CI was determined by screening 52 individual colonies recovered from XLD plates for antibiotic resistance by patching on LB plates containing the appropriate antibiotic, thus the limit of detection on these experiments is less than 100-fold.

For the single-infection Swiss Webster experiments, streptomycin-treated Swiss-Webster mice were inoculated with a single bacterial strain at 10^7^ CFU. Where indicated, 100 μL of 1 M F-Asn was administered intragastrically (i.g.) daily and/or a probiotic strain was administered in a 1:1 mixture with the tested *Salmonella* strain. Ceca and feces were harvested on day four post-infection. CFU enumeration was determined by plating on media selective for WT (LB cam) and the mutant (LB kan). For the *fraB* E214A mutant, which is not marked, CFU enumeration was determined by plating on XLD.

For experiments involving the CBA/J mice on a high-fat chow, CBA/J mice were switched to this diet (Bioserv S3282) three days prior to inoculation with a single *Salmonella* strain, either JLD1214 or ASD4355, at 10^9^ CFU (day 0). Where indicated, the mice were also inoculated with either 10^9^ CFU of ASD203 or *E. coli* Nissle on day 0, and 100 μL of 1 M F-Asn was administered intragastrically on days 0–3. *Salmonella* strains JLD1214 and ASD4355 were enumerated from feces or cecum by plating on LB cam while ASD203 was enumerated by plating on LB kan. Lipocalin was measured from feces as previously described [6].

### Limiting dilution series

Cecal contents from a 1:1 competition of WT and *fraB80*::kan mutant were used to calculate the percentage of *fraB* mutants that had become resistant to F-Asn intoxication *in vivo*. The cecum of each infected mouse was homogenized and serially diluted. One dilution series was plated on LB kan plates to determine the total number of *fraB80*::kan mutants present. The other dilution series were performed in 96-well plates in medium selective for *fraB80*::kan mutants that have become resistant to F-Asn (M9 medium supplemented with 5 mM glucose, 5 mM F-Asn, and 50 μg/ml kanamycin). Each plate was incubated at 37°C overnight. After growth overnight, the lids were removed, and the absorbance was read at 600 nm in the Spectra Max M2 (Molecular Devices). The frequency at which a particular dilution yielded growth was used to determine the percentage of resistant mutants present: percentage mutant = -ln(frequency that growth was not obtained)/total bacteria per well. For example, if 3 of 10 wells had no growth at a dilution where there were 240 bacteria per well, we determined that 0.5% of the *fraB80*::kan mutants were F-Asn resistant.

*Mutation fluctuation tests-* To calculate the mutation rate to F-Asn resistance, 94 colonies of *fraB80*::kan were inoculated into the wells of a 96-well plate containing 1X M9 medium supplemented with 5 mM glucose and 5 mM F-Asn. One well was inoculated with wild-type as a positive growth control and one well was inoculated with wild-type but the growth medium was supplemented with chloramphenicol as a negative control for growth. Three of the wells were immediately titered by serial dilution and drip plated on LB plates to enumerate the starting number of CFU in each well. The plate was lidded and incubated at 37ºC for 16 h. After incubation, three of the wells were titered by serial dilution and drip plated on LB plates to enumerate the total CFU in each well after incubation. Then, the lid was removed and the absorbance was read at 600 nm in a Spectra Max M2 plate reader (Molecular Devices). The mutation rate in events per culture (m) = -ln(# of wells with no growth/total number of wells). This rate was then multiplied by the number of cell divisions per culture to obtain the mutation rate per cell division using the method of Luria and Delbrück [47].

### Isolation of transposon mutants resistant to F-Asn

A mutant library was created by transducing a T-POP transposon [Tn*10*dTet[del25] [48]] into the *fraB*80::kan mutant background, HMB206. To prepare the lysate, *Salmonella enterica* serovar Typhimurium strain TH3923 was grown overnight in LB at 30°C. The cells were washed and subcultured to OD_550_ of ~0.4. One mL of 0.5 mg/mL mitomycin C was added to induce phage production, and the cells were allowed to lyse with shaking at 37°C for 4 h. The solution was filter sterilized, transferred to a glass tube, and 100 μL of chloroform was added. The transduction was performed immediately after lysate preparation. A 5-mL culture of HMB206 carrying pNK2880, a plasmid that encodes a Tn*10* transposase with relaxed target specificity, was grown in LB with ampicillin at 37°C overnight with shaking. In a sterile flask, 5 mL of the TH3923 lysate was added to 5 mL of the HMB206 + pNK2880 culture, and incubated for 25 min at 37°C. The cells were washed twice in LB with EGTA and resuspended in 50 mL of LB with EGTA for a 60-min outgrowth. At this point, the number of mutants in the culture was determined by titering on LB with tetracycline. Tetracycline was then added to the remainder of the culture and the culture allowed to grow at 37°C overnight after which glycerol stocks were created and stored at −80°C. The library contained 25,000 mutants.

A selection was applied to the library to obtain mutants that could grow in the presence of F-Asn. Ninety-six mutants were obtained using a 96-well format in which 2 μL of library was inoculated into 200 μL of medium and one mutant was isolated from each well after overnight growth at 37 °C. Growth of all mutants in M9 + glucose + F-Asn was confirmed. Inverse PCR was used to amplify the transposon insertion site. Genomic DNA of the mutant was purified using the GenElute bacterial genomic DNA kit (Sigma). The genomic DNA was digested with the restriction enzyme *Nla*III (NEB). The reaction was incubated at 37°C for 3 h, and then the enzyme was heat inactivated at 65°C for 20 min. The digested DNA was self-ligated to form circular DNA using T4 DNA ligase at 15°C overnight (NEB). The ligation was purified with a QIAprep® Spin Miniprep Kit (Qiagen). The DNA was then digested with *Dra*I for 3 hours at 37°C and then the enzyme heat inactivated for 20 min at 65°C (NEB). PCR was performed with primers IPCRF and IPCRR [49]. The PCR product was purified with QIAquick® PCR Purification Kit (Qiagen) and submitted for Sanger sequencing to the Plant-Microbe Genomics Facility at the Ohio State University.

### Histopathology

Samples of the proximal colon were removed from mice, and a portion was immersion fixed in 10% (v/v) neutral buffered formalin, processed by routine methods, and embedded in paraffin wax by the Comparative Pathology and Mouse Phenotyping Shared Resource (CPMPSR) at the Ohio State University. Sections (4 μm) were stained with hematoxylin and eosin (H&E) as previously described [39] and scored in a blinded fashion by a veterinary pathologist, board certified by the American College of Veterinary Pathologists (ACVP).

### Animal assurance

All animal work was performed using protocols approved by our Institutional Animal Care and Use Committee (IACUC; OSU 2009A0035) and in accordance with the relevant guidelines set forth in the PHS “Guide for the Care and Use of Laboratory Animals”.

## Results

### Alleles of *fraB* have different phenotypes with regard to F-Asn sensitivity

**Fig 2:**
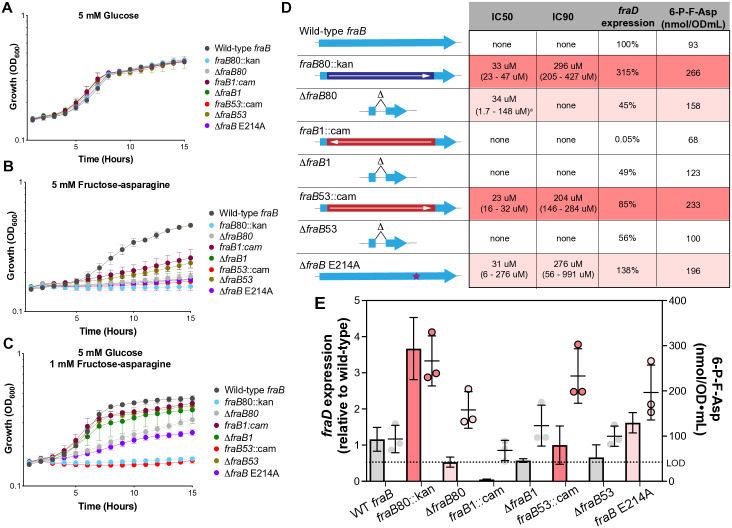
Different alleles of *fraB* exhibit different growth phenotypes, altered *fraD* expression levels, and variable 6-P-F-Asp build-up. Growth of different *fraB* mutants in M9 minimal medium containing **(A)** 5 mM glucose, **(B)** 5 mM F-Asn, or **(C)** 5 mM glucose and 1 mM F-Asn. **(D)** A diagram of the different *fraB* alleles indicating the antibiotic cassette, and its orientation, used to replace *fraB*, or the deletion of the antibiotic cassette, or the location of the point mutation E214A (not to scale). The IC_50_ and IC_90_ values are listed for all *fraB* mutants, along with the respective 95% confidence interval. The expression of *fraD* relative to wild-type, and the concentration of 6-P-F-Asp for each strain are also shown. The *fraD* expression and 6-P-F-Asp measurements were collected from the same cultures that were grown for 4 h in M9 minimal medium supplemented with 5 mM glucose, at which point F-Asn was added to 1 mM and incubation continued for 30 min. qRT-PCR data were obtained from two biological replicates, each with two technical replicates. 6-P-F-Asp concentrations (expressed as nanomoles per volume cells * the optical density of the culture, or nmol/OD ml) were determined from three biological replicates, each with two technical replicates. “None” indicates that inhibition of growth was not observed at concentrations of F-Asn up to 5 mM. ^a^ A 95% confidence interval could not be calculated for this strain, so a 50% confidence interval was used instead. Color gradient from white to red indicates level of growth inhibition observed. **E)** Graphical representation of the relative *fraD* expression and 6-P-F-Asp concentration for each strain (data from panel **D)**. Dotted line is the limit of detection line (LOD) at 42 nmol per OD*mL of cells which was measured based on a calibration curve and area under the curve analysis for standard samples. Color gradient from white to red indicates level of growth inhibition observed.

We previously reported that the growth of a *fraB80*::kan mutant of *Salmonella* is inhibited by the presence of F-Asn with an IC_50_ of 19 μM [30]. Here, we report that the growth of a *fraB1*::cam mutant of *Salmonella* is unaffected by the presence of F-Asn (Fig 2). We hypothesize that the *fraB1*::cam mutation is polar on *fraD* expression, while the *fraB80*::kan mutation is not. Since *fraD* expression is required for synthesis of the toxic metabolite, 6-P-F-Asp, expression of *fraD* would be required for F-Asn to inhibit the growth of a *fraB* mutant. Both mutations replace nearly all of *fraB*, except for ten codons each at the 5’ and 3’ termini. The kan cassette is oriented in the same orientation as *fraB*, while the cam cassette is oriented in the opposite direction (Fig 2). Although neither cassette has a Rho-independent terminator of transcription, the kan (but not cam) promoter may increase *fraD* expression above wild-type levels. This possibility prompts the question of how much growth inhibition would be observed if expression of the operon was not altered. This question is relevant to drug discovery efforts, as it is essential to determine if a putative small molecule inhibitor of FraB would inhibit the growth of wild-type *Salmonella* in the presence of F-Asn [50–52]. To address this key question, we utilized multiple strategies.

First, we utilized a *fraB* E214A mutation in the native *fra* locus [27]. This mutation should have no polar effects on *fraD* expression. Additionally, the FraB E214A mutant is catalytically inactive, making it a good proxy for a wild-type cell in the presence of a presumptive drug targeting FraB [27,50–52]. Second, we constructed a strain in which the cam cassette was inserted in the same orientation as *fraB* (*fraB53*::cam). Finally, we also made strains in which the cam and kan cassettes were removed by Flp-mediated recombination (Δ*fraB1*, Δ*fraB53*, and Δ*fraB80*). For each strain, we then measured *fraD* expression, the accumulation of 6-P-F-Asp, and growth in the presence of F-Asn (Fig 2).

The *fraD* gene is most highly expressed, 6-P-F-Asp is at its highest concentrations, and growth is the most severely inhibited, in the *fraB80*::kan and *fraB53*::cam mutants (Fig 2). In contrast, the lowest expression of *fraD*, the lowest concentrations of 6-P-F-Asp, and the least growth inhibition are observed with the *fraB1*::cam, Δ*fraB1*, and Δ*fraB53* mutants. The *fraB* E214A and the Δ*fraB80* mutants had *fraD* expression similar to wild-type. 6-P-F-Asp accumulation and growth inhibition were also intermediate in these mutants compared to the other strains. Although the IC_50_ of F-Asn for the E214A mutant is similar to that of the *fraB80*::kan mutant, the magnitude of the inhibitory effect is lower based on growth *in vitro*. The expression of *fraD* in the *fraB* E214A or Δ*fraB* mutants is driven by the native *fraB* promoter, while *fraD* expression is increased in the *fraB80*::kan mutant, presumably by the kan promoter. Despite the seemingly strong correlations, not all the results are easily interpreted.

First, similar to the *fraB80*::kan strain, the *fraB53*::cam mutant is severely intoxicated by F-Asn and consistently shows high 6-P-F-Asp accumulation but *fraD* expression is not higher than the wild-type. It is also not clear why the Δ*fraB1* and Δ*fraB53* mutants fail to accumulate as much 6-P-F-Asp as the Δ*fraB80* mutant. It is likely that there are aspects of FraD (post-transcriptional) regulation that remain to be elucidated. Second, the *fraB53*::cam but not the *fraB1*::cam mutant is highly intoxicated by F-Asn (Fig 2). The difference in these mutations is the orientation of the cam cassette. There is intoxication by F-Asn only when the cassette is oriented with *fraB* and not opposite to *fraB*. When oriented opposite to *fra,* there is a stop codon created in the *fraB* gene that is likely polar on *fraD* (i.e., premature termination of transcription due to uncoupling of translation and transcription); indeed, *fraD* is not expressed in *fraB1*::cam (Fig 2). Without FraD, there can be no synthesis of the toxic intermediate, 6-P-F-Asp, explaining the absence of intoxication in *fraB1*::cam. In the *fraB80*::kan mutant, the kan cassette is oriented with *fraB,* and *fraD* expression is three-fold higher than wild-type, providing an explanation for the enhanced intoxication of this strain by F-Asn (Fig 2).

### Competition experiments to determine the fitness of a *fraB* E214A mutant in mouse models

**Fig 3:**
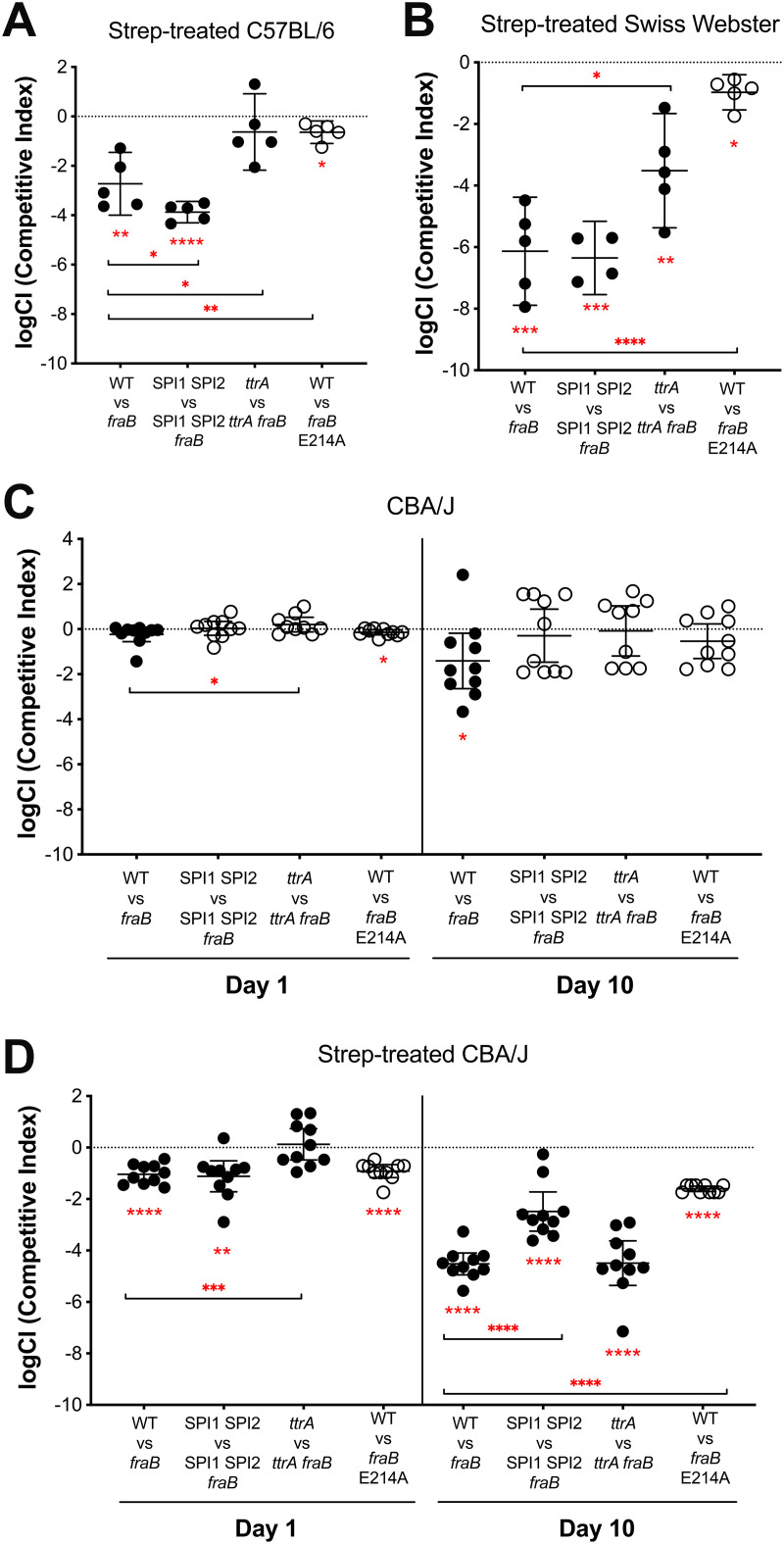
The *Salmonella fraB* E214A mutant is attenuated in different murine models. In each experiment, a 1:1 mixture of two different bacterial strains is inoculated intragastrically (i.g.) and then recovered later from ceca to determine the competitive index (CI) of the two strains. The pairs of strains inoculated are listed on the X-axis. The use of strain JLD1214 (wild-type 14028 marked with a chloramphenicol-resistance cassette in a neutral location [24,30,39,53,54]) is indicated as “WT”. The use of strain HMB206 (*fraB80*::kan) is indicated as “*fraB*”. The use of strain ASD1312 (*fraB* E214A) is indicated as “*fraB* E214A”. **A)** Streptomycin-treated C57BL/6 mice inoculated with 10^7^ total CFU and harvested after four days. **B)** Streptomycin-treated Swiss-Webster mice inoculated with 10^7^ total CFU and harvested after four days. **C)** CBA/J mice inoculated with 10^9^ total CFU and harvested after either one or ten days. **D)** Streptomycin-treated CBA/J mice inoculated with 10^7^ total CFU and harvested after four days. Black circles indicate that the CI was determined by plating on media selective for WT (LB cam) or the mutant (LB kan). Open circles indicate that the CI was determined by screening 52 individual colonies recovered from XLD plates for antibiotic resistance by patching on LB plates containing the appropriate antibiotic, thus the limit of detection on these experiments is less than 100-fold. The data were transformed and the mean with standard deviation is shown. Statistical significance of logCI being different than zero was determined using a one sample t-test (two-tailed). Statistical significance of two competitions being different from each other was determined using a student’s t-test (unpaired, two-tailed). * = *P* value <0.05, ** = *P* value <0.005, *** = *P* value <0.0005, **** = *P* value <0.00005.

The intermediate level of growth inhibition observed with the *fraB* E214A mutant in the presence of F-Asn suggested that this mutant may not be as attenuated in mice as the *fraB80*::kan mutant. This notion was confirmed in several mouse models using competition experiments in which the wild-type was inoculated with a mutant in a 1:1 ratio (Fig 3). If the mutant is recovered in less than a 1:1 ratio, it is considered less fit than the wild-type. Initial studies were performed in a streptomycin-treated C57BL/6 mouse model. In this model, the *fraB* E214A mutant has a 4-fold decrease in fitness compared to wild-type, while the *fraB80*::kan mutant has a 524-fold decrease in fitness (Fig 3A). Similarly, in the streptomycin-treated Swiss Webster mouse model, the *fraB* E214A mutant is significantly less attenuated than the *fraB80*::kan mutant (9-fold vs > 10,000-fold, respectively) (Fig 3B).

We next chose to test the *fraB* mutant phenotypes in a CBA/J model. In this model, the gastrointestinal tract slowly becomes inflamed in the presence of *Salmonella* (8–12 days post-infection) without the need for perturbation of the normal microbiota with antibiotics [34,35]. At 10 days post-infection, the *fraB80*::kan mutant was significantly attenuated (25-fold) while the *fraB* E214A mutant was not (Fig 3C). However, the presence of only the wild-type or the mutant in 50% of the mice suggested that the bacterial population was undergoing a population bottleneck. To circumvent this issue, we repeated the experiment but pre-treated the mice with streptomycin. In streptomycin-treated CBA/J mice, both the *fraB80*::kan and the *fraB* E214A mutants are equally attenuated one day post-infection (10- and 8-fold, respectively) (Fig 3D). After 10 days post-infection, the *fraB80*::kan mutant was no longer recovered. Comparison to the number of wild-type recovered indicates that the *fraB80*::kan mutant is at least 10,000-fold attenuated. The *fraB* E214A mutant was not recovered either, but since the *fraB* E214A mutant is not marked with an antibiotic resistance gene, *Salmonella* was first recovered on XLD plates and then 52 individual colonies from each mouse were patched on LB cam, with growth indicating the strain was wild-type and no growth indicating the strain was a *fraB* E214A mutant. The limit of detection in this experiment is low, but the *fraB* E214A mutant was nonetheless not recovered.

### The roles of SPI1 SPI2, and *ttrA*

*Salmonella* benefits from SPI1 and SPI2, each encoding a type III secretion system, as they provoke an inflammatory response that disrupts the microbiota [10]. The disruption of the microbiota provides access to nutrients including 1,2-propanediol, ethanolamine, glucarate, galactarate, L-lactate, and F-Asn [7,8,31,55–57]. Unique access to nutrients is beneficial, but gaining increased energy over the competition (e.g., through respiration of metabolites) is even more advantageous from a pathogen expansion perspective. The oxidative burst that accompanies inflammation leads to the accumulation of tetrathionate, nitrate, and even oxygen, all of which are used as terminal electron acceptors by *Salmonella* for respiration [56,58–60]. The *ttrA* gene is required for *Salmonella* to utilize one of these electron acceptors, tetrathionate [59].

In 2014, we tested and believed that we had confirmed the requirement of SPI1 and SPI2 in order for *Salmonella* to gain access to F-Asn [24]. However, the studies performed here reveal an error in that paper [24]. In our earlier study, we demonstrated that two independently constructed *fraB:*:kan mutants were attenuated in a variety of mouse models that are susceptible to inflammation, and not attenuated in mouse models that are not susceptible to inflammation. Both mutants had fitness restored by complementation with a plasmid encoding the *fra* island. Those results are correct. However, because SPI1 and SPI2 are required for *Salmonella* to induce inflammation of the intestine, we then tested the hypothesis that in the absence of SPI1 and SPI2, a *fraB* mutant would no longer gain access to F-Asn and be intoxicated. Therefore, a competition experiment was performed between a SPI1 SPI2 double mutant and a SPI1 SPI2 *fraB* triple mutant in germ-free or strep-treated C57BL/6 mice. Both strains were recovered in equivalent numbers indicating that the *fraB* mutation does not cause attenuation in the SPI1 SPI2 mutant background [24]. Unfortunately, we used a *fraB1*::cam mutation to construct the SPI1 SPI2 *fraB* triple mutant because the SPI2 locus had already been disrupted with a kan cassette [24]. Since we now know that the *fraB1*::cam mutation is not intoxicated by F-Asn, there was no toxicity for the SPI1 and SPI2 mutations to alleviate in the SPI1 SPI2 *fraB* triple mutant. (Incidentally, the figure in that paper [24] is mislabeled and indicates that it was a *fraB*::kan mutation in the SPI1 SPI2 strains, although the figure legend correctly states that it was a *fraB1*::cam mutation.)

We have repeated the experiments here, placing a *fraB80*::kan mutation into a SPI1 SPI2 background. In competition experiments using CBA/J mice at ten days post-infection, the *fraB80*::kan mutant was attenuated 25-fold compared to wild-type, while a *fraB80*::kan SPI1 SPI2 triple mutant was not attenuated compared to a SPI1 SPI2 double mutant (Fig 3C). While this finding would appear to confirm that a *fraB* mutation does not cause attenuation when present in a SPI1 SPI2 background, it is not an unambiguous result as the SPI1 SPI2 strains appeared to undergo a population bottleneck in these mice, with roughly half of the mice containing only *fraB* mutant bacteria and half of the mice containing only *fraB*^+^ bacteria at the end of the experiment. This bottleneck is likely due to both strains in the competition lacking SPI1 and SPI2 and failing to induce inflammation. When the CBA/J mice were pre-treated with streptomycin, the bottleneck effects were ameliorated and the *fraB80*::kan mutant was greatly attenuated compared to wild-type (>30,000-fold). Moreover, the *fraB80*::kan SPI1 SPI2 triple mutant was attenuated, but not as much as the SPI1 SPI2 double mutant (251-fold) (Fig 3D). Thus, it appears that the SPI1 and SPI2 mutations at least in CBA/J mice partially abrogate the *fraB80*::kan phenotype. Since inflammation eliminates competitors for F-Asn [7], we hypothesize that the SPI1 SPI2 mutants fail to provoke inflammation and leave intact competitors for F-Asn. Therefore, there is less F-Asn is available to intoxicate the *fraB* mutant. Interestingly, in strep-treated C57BL/6 mice and strep-treated Swiss-Webster mice, the SPI1 and SPI2 mutations did not abrogate the *fraB80*::kan phenotype at all (Fig 3A, 3B). This outcome is likely attributable to the fact that streptomycin treatment eliminates competitors for F-Asn, thereby allowing the *fraB* mutant to acquire F-Asn and to be intoxicated by 6-P-F-Asp. Thus, SPI1 and SPI2 are essential for *fraB* phenotypes in some scenarios but not others presumably because access to F-Asn is being regulated by other factors. These results also emphasize how different mouse models could result in different conclusions as to the essentiality of specific genes for *Salmonella* pathogenesis.

We also confirmed and extended our previous studies on the dependence of the *fraB* phenotype on *ttrA*, a gene required for the utilization of tetrathionate as an electron acceptor (Fig 3) [8,24,61,62]. Confirming our previously published result, the *fraB* phenotype is dependent on *ttrA* in a streptomycin-treated C57B/6 mouse model (Fig 3A) [24]. Additionally, we determined that the *ttrA1* mutation partially abrogates the attenuation of a *fraB80*::kan mutant in streptomycin-treated Swiss webster mice (Fig 3B). Interestingly, in a streptomycin-treated CBA/J model, tetrathionate utilization is required for a *fraB80*::kan phenotype at one day post-infection but not at 10 days post-infection when a *fraB80*::kan *ttrA1* double mutant is just as attenuated as the *fraB80* single mutant (Fig 3D). The mechanism by which *ttrA* affects the intoxication of a *fraB* mutant by F-Asn is not clear.

### Acquisition of resistance of a *Salmonella fraB* mutant to F-Asn

An important aspect of any therapeutic strategy is to understand the mechanism(s) by which cells can become resistant. To determine the mechanisms by which a *Salmonella fraB80*::kan mutant may become resistant to F-Asn, we performed a selection for resistant mutants. A library of 25,000 T-POP mutations was created in the *Salmonella fraB80*::kan strain HMB206. The T-POP transposon is a mTn*10* derivative that lacks a transcription terminator at the end of the *tetA* gene encoding the tetracycline efflux pump [48]. This design allows transcription to proceed into neighboring genes, albeit under the control of tetracycline. Thus, in addition to the disruption of genes, artificial activation can be achieved. Selection was applied to the library by growing the cells in M9 minimal medium containing 5 mM glucose, 5 mM F-Asn, kanamycin and tetracycline. To avoid the characterization of siblings, the library was grown in the wells of a 96-well plate and one mutant was obtained from each well. The insertion site was determined for 96 mutants resistant to F-Asn. Of 96 sequences, nine mapped to *fraD*, 86 mapped to *fraA* and one mapped to *narU*. When the *narU* mutation was transduced into HMB206 (a clean background), it no longer conferred resistance to F-Asn. This finding indicates *narU* is not responsible for the F-Asn-resistance phenotype and that there was a second mutation in the original strain (which we did not map). Thus, in this genetic screen, only *fraD* and *fraA* conferred resistance to F-Asn. Interestingly, *fraE* mutations were not isolated, which is consistent with our findings that the *ansB* gene encodes an asparaginase that can partially compensate for the lack of *fraE* [26].

We determined the spontaneous mutation rate to F-Asn resistance (no transposons, just natural mutation rate) using a fluctuation test in which 94 separate cultures of a *fraB80*::kan mutant were grown in the presence of 5 mM F-Asn. The number of cultures that yielded no resistant mutants was used to calculate the mutation rate using the method of Luria and Delbrück [47]. The rate of spontaneous mutation to F-Asn resistance was determined to be 6.5 x 10^−8^ mutants per cell division. This rate is similar to the rate that Luria and Delbrück obtained when selecting for resistance to T1 phage and is expected as simple knock-outs confer resistance in each system (i.e., *tonB* or *fhuA* for T1^r^, and *fraA* or *fraD* for F-Asn^r^).

**Fig 4:**
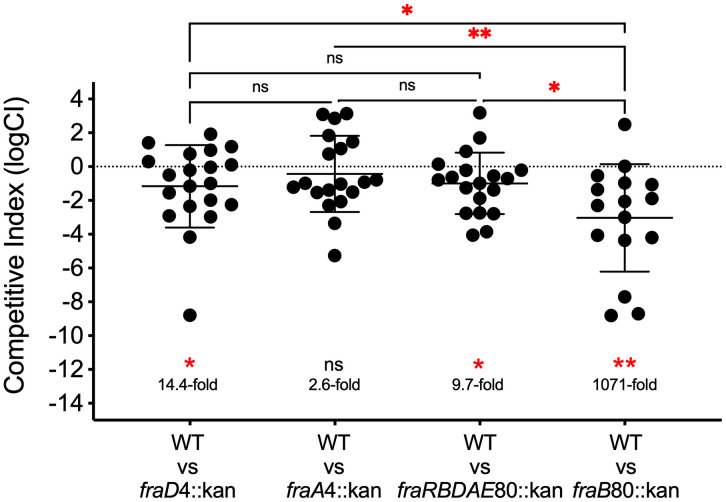
A *fraD* mutant and a mutant lacking the *fra* island are slightly attenuated in a CBA/J mouse model. In each experiment, 6 to 10-week-old CBA/J mice were inoculated intragastrically (i.g.) with a 1:1 mixture of two different bacterial strains (10^9^ total CFU) and then recovered from ceca 15 days later to determine the competitive index (CI) of the mutant. The pairs of bacterial strains inoculated are listed on the X-axis. The wild-type strain is JLD1214 which is 14028 marked with a neutral chloramphenicol resistance cassette. The *fraD4*::kan strain is HMB184, the *fraA4*::kan strain is HMB247, the *fraRBDAE80*::kan is HMB205, and the *fraB80*::kan strain is HMB206. **B)** The competitive index data from panel A of *fraD*4::kan, *fraA*4::kan and *fraRBDAE*80::kan combined. The CI was determined by plating on media selective for WT (LB cam) or the mutant (LB kan). The data were transformed and the mean with standard deviation is shown. Statistical significance of a logCI being different than zero was determined using a one sample t-test (two-tailed). Statistical significance of two competitions being different from each other was determined using a student’s t-test (unpaired, two-tailed). * = *P* value <0.05, ** = *P* value <0.005.

When a wild-type (JLD1214) and *fraB80*::kan mutant (HMB206) are inoculated into CBA/J mice in a 1:1 ratio (10^9^ CFU), the wild-type is recovered in 1000-fold higher numbers than the mutant after 16 days (Fig 4). We were curious as to why *fraB80*::kan mutants were recovered given the evidence that F-Asn is bactericidal to the *fraB80*::kan mutant during infection of mice [30]. We hypothesized that the *fraB80*::kan mutants may be acquiring secondary mutations (presumably in *fraA* or *fraD*) that make them resistant to F-Asn. To test this hypothesis, we performed a limiting-dilution experiment on recovered homogenized ceca from ten infected mice to determine the percentage of *fraB80*::kan mutants that had become resistant to F-Asn. In four of the mice, all of the *fraB80*::kan mutants recovered had become resistant to F-Asn. In two mice, none of the *fraB80*::kan mutants recovered had become resistant to F-Asn. Three mice yielded intermediate values in which 0.4%, 0.4% and 50% of the *fraB80*::kan mutants recovered were resistant to F-Asn. No *fraB80*::kan mutants were recovered at all from the tenth mouse so a percentage could not be obtained. In conclusion, *fraB80*::kan mutants often become resistant to F-Asn in this mouse model.

Since suppressor mutations that arise *in vivo* render a *fraB80*::kan mutant resistant to F-Asn, we tested the hypothesis that these mutants might still be attenuated due to a lack of F-Asn utilization. We have previously tested this idea using a *fraD* mutant and a *fra* island mutant (lacking all of *fraR-fraBDAE*) and found that neither were attenuated in a strep-treated Swiss Webster mouse model [30]. Here, we re-examined this possibility using larger numbers of CBA/J mice. We tested a *fraD* mutant, *fra* island mutant, and a *fraA* mutant. We found that in competition with wild-type (JLD1214), the *fraA* mutant was 2.6-fold attenuated, the *fraD* mutant was 14-fold attenuated, and the *fra* island mutant was 9-fold attenuated (Fig 4). Thus, any suppressor mutant that can no longer utilize F-Asn should be at least slightly attenuated in CBA/J mice over time.

### Use of single infections to determine the amount of inflammation caused by a *Salmonella fraB* mutant

**Fig 5:**
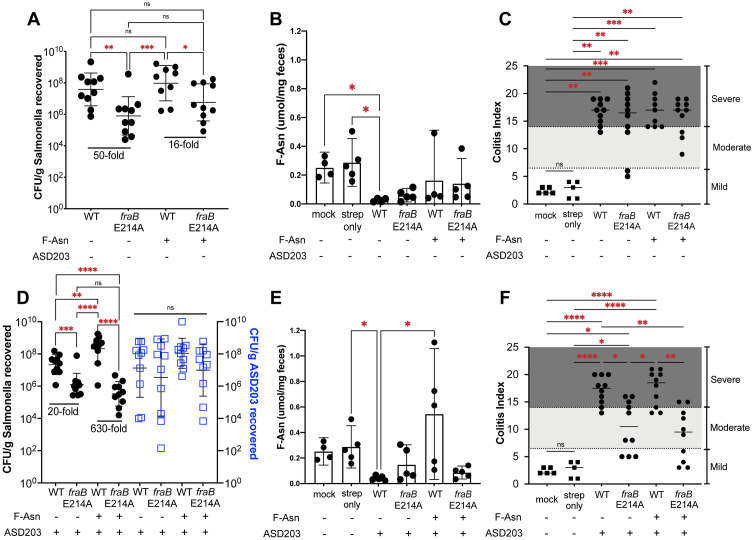
A *fraB* E214A mutant is attenuated in a single infection streptomycin-treated mouse model. Streptomycin-treated Swiss Webster mice were infected with the indicated strains. On day 4 post-infection, CFU were enumerated **(A, D)**, F-Asn in feces was measured by LC-MS/MS **(B, E)**, and histopathology scores of proximal colon were determined **(C, F)**. Mock samples are mice that were not inoculated with *Salmonella* and “strep-only” mice are mice that received only streptomycin-treatment. Some groups were administered 29.4 mg of F-Asn intragastrically (i.g.) daily. For panels A-C the wild-type is JLD1214 which is 14028 marked with a chloramphenicol resistance cassette in a neutral location in the chromosome, and the *fraB* E214A mutant is ASD1312. For panels D-F the wild-type is JLD1214 and the *fraB* E214A mutant is ASD4355 which is marked with a chloramphenicol resistance cassette in a neutral location in the chromosome. Where indicated, the probiotic strain ASD203 was inoculated i.g. on day 0 (10^9^ CFU). Panel A shows the results from two separate experiments (5 mice per group in each experiment, 10 mice per group total). The inoculum was 1.3 x 10^8^ and 8.4 x 10^7^ for the wild-type and 9.8 x 10^7^ and 7.2 x 10^7^ for the *fraB* E214A mutant. The CFU recovered of the wild-type strain was determined by plating on LB cam, while the number of *fraB* mutant recovered was determined by plating on XLD. Panel D shows the results from two separate experiments (5 mice per group in each experiment, 10 mice per group total). The inoculum was 1.2 x 10^8^ and 5 x 10^7^ for the wild-type and 1.5 x 10^8^ and 2.8 x 10^7^ for the *fraB* E214A mutant. When probiotic ASD203 is present, CFU of each strain was determined by plating on media selective for WT (LB cam), the mutant (LB cam), or the probiotic (LB kan). Probiotic counts are indicated by the blue squares. The data were transformed and the mean with standard deviation is shown. Statistical significance of two groups being different from each other was determined using an ordinary one-way ANOVA. * = *P* value <0.05, ** = *P* value <0.005, *** = *P* value <0.0005, **** = *P* value <0.00005.

We have typically measured the fitness of *fraB* mutants during competitive infections with the wild-type given their higher precision compared to single and separate infections of mice with these strains. However, we recognize that the presence of wild-type *Salmonella* during competitive infections would contribute additively to inflammation and thereby help the *fraB* mutant gain access to F-Asn [7]. If a *fraB* mutant were to be used alone, SPI1 and SPI2 in the *fraB* mutant should induce inflammation thus engendering F-Asn availability but the F-Asn would soon after intoxicate the *fraB* mutant. If inflammation subsides upon elimination of the *fraB* mutant, the availability of F-Asn may also decline and promote a revival of the remaining *fraB* mutant cells. This oscillation triggered by the undulating inflammation and F-Asn prompts the question of whether the need for inflammation (a pre-requisite for F-Asn) be offset by adding exogenous F-Asn. To answer this question, we infected streptomycin-treated Swiss Webster mice with wild-type alone or the *fraB* E214A mutant alone. Four days later, the ceca were harvested for enumeration of bacteria, the proximal colon was collected for histopathology, and fecal samples were collected for determination of F-Asn concentration. The *fraB* E214A mutant was recovered 48-fold lower than the wild-type, however, the inflammation induced by the *fraB* mutant was similar to that induced by the wild-type (Fig 5). Surprisingly, providing exogenous F-Asn did not substantially change the results.

### Probiotics enhance the attenuation of *fraB* E214A in streptomycin-treated Swiss Webster mice

*E. cloacae* can enhance the attenuation of a *fraB* mutant in germ-free mice [24]. Since *E. cloacae* cannot utilize F-asn [32], we hypothesize that this enhancement is due to *E. cloacae* competing with *Salmonella* for a large proportion of the nutrients available, driving *Salmonella* to consume F-Asn as a higher proportion of its nutrient pool. We decided to re-visit this idea, using what we refer to as our “*Salmonella* probiotic” strain, ASD203, which lacks SPI1 and SPI2 and therefore cannot induce inflammation [10,39]. Additionally, this strain cannot consume F-Asn due to a deletion of the entire *fra* island (*fraR-fraBDAE*). The *ansB* gene is also deleted as this enzyme is capable of converting F-Asn to F-Asp [26]. The addition of this “probiotic” should provide a competitor to *Salmonella* for all nutrients except F-Asn, effectively forcing the use of F-Asn as a nutrient. The probiotic strain was added in a 1:1 ratio with either wild-type or *fraB* E214A mutant *Salmonella*, in the presence or absence of exogenous F-Asn. The number of *fraB* mutant bacteria recovered, and the resulting inflammation, were dramatically reduced by the probiotic, regardless of whether additional F-Asn was provided (Fig 5).

### A high fat diet eliminates the need for streptomycin pretreatment in a Salmonella gastroenteritis model

**Fig 6:**
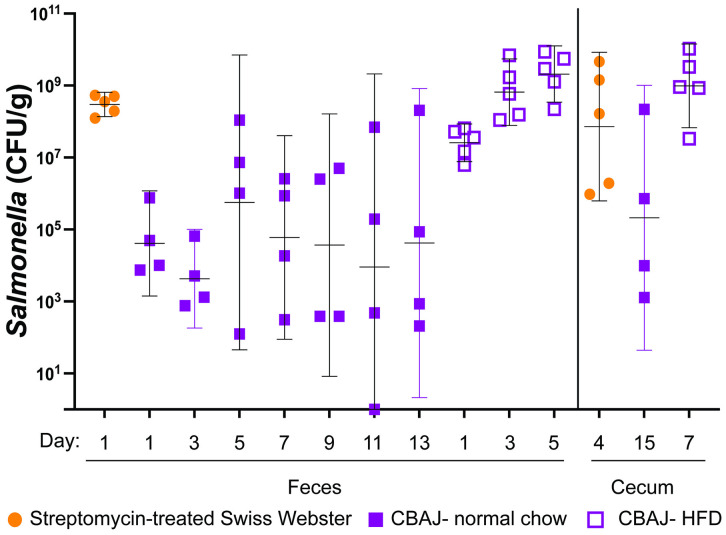
Optimization of a diet-enhanced colitis mouse model. Three different mouse models were compared for recovery of wild-type *Salmonella* (JLD1214), after oral inoculation, from feces or cecum, as indicated. Swiss-Webster mice treated with streptomycin were orally inoculated with 1 x 10^7^ CFU; CBA/J mice on normal chow were inoculated with 1 x 10^9^ CFU; and CBA/J mice that had been on high-fat diet for ten days were inoculated with 1 x 10^9^ CFU. The data was transformed and the mean with standard deviation is shown. Statistical significance of two groups being different from each other was determined using an ordinary one-way ANOVA. * = *P* value <0.05, ** = *P* value <0.005, *** = *P* value <0.0005, **** = *P* value <0.00005.

Mice are quite resistant to *Salmonella*-mediated inflammation of the gastrointestinal tract unless the microbiota is first disrupted with streptomycin (or other antibiotics) [63–68]. These streptomycin-treated mouse models have been extremely useful for studying *Salmonella*-mediated inflammation. However, the use of streptomycin is not ideal when the research objective is to study the interaction of *Salmonella* with an intact or “normal” microbiota. The CBA/J mouse model solves this problem in that *Salmonella* can persist in these mice and given enough time (8–12 days) begin to cause inflammation of the gastrointestinal tract and thrive in this inflamed environment. A disadvantage of the CBA/J model is that only about one third of the mice become “high responders” with a high *Salmonella* burden and inflammation. The remaining mice tend to have a low *Salmonella* burden and no inflammation. This heterogeneity leads to high variability. Recent reports [69,70] that a high-fat diet can increase susceptibility of mice to *Salmonella*-mediated inflammation prompted us to test the hypothesis that the percentage of CBA/J mice that become high responders could be increased by placing them on a high-fat diet. This idea was confirmed when we compared the recovery of *Salmonella* from orally inoculated CBA/J on normal chow versus high-fat diet. The CFU recovered from the mice on a high-fat diet were much more consistent and all mice were high responders (Fig 6).

**Fig 7:**
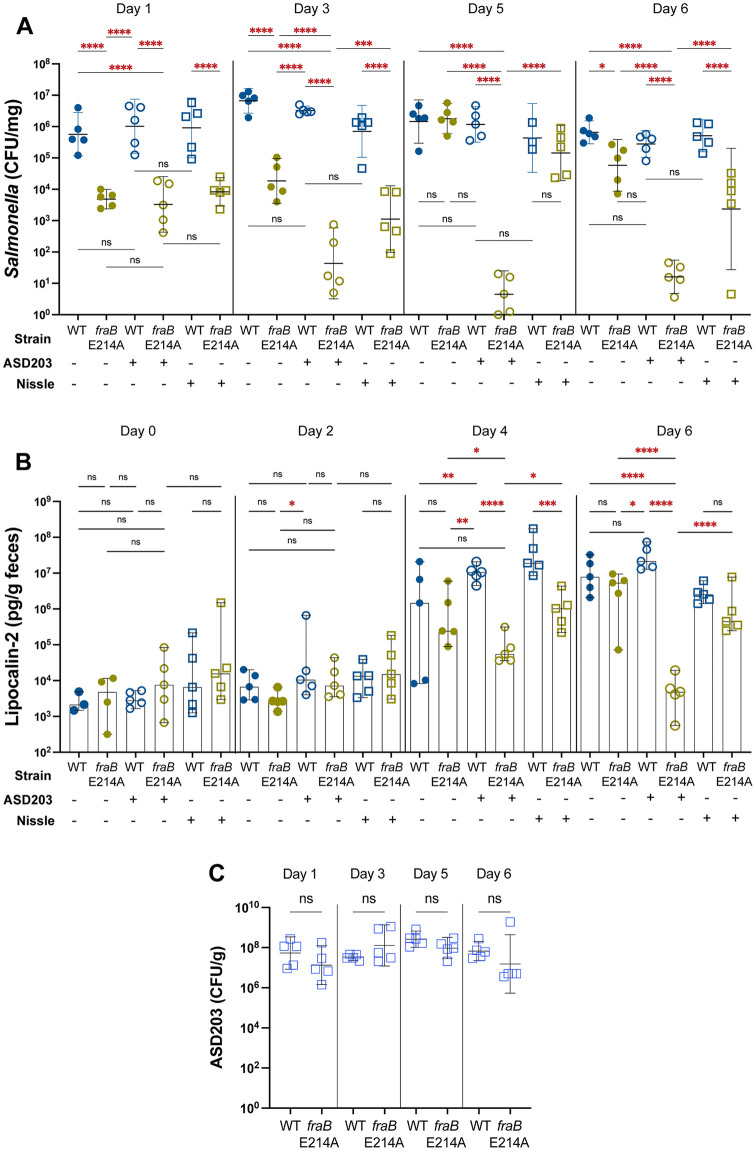
A combination of F-Asn and a probiotic can reduce the bacterial burden and inflammation caused by a *Salmonella fraB* E214A mutant during infection of CBA/J mice on a high fat diet. Groups of five female CBA/J mice were placed on a high-fat diet, *ad libitum*, 3 days prior to oral inoculation with either 1 x 10^9^ CFU of wild-type (JLD1214) or a *fraB* E214A mutant (ASD4355). The inoculation of these *Salmonella* strains marks day 0 and is indicated on the X-axis. Also on day 0, where indicated, the mice were orally inoculated with 1 x 10^9^ CFU of a probiotic strain, either ASD203 or *E. coli* Nissle, as indicated below the X-axis. On days 0-3, all mice were orally gavaged with 100 μl of 1 M F-Asn. **A)** On days 1, 3, and 5 *Salmonella* were enumerated in feces. On day 6, the mice were sacrificed and *Salmonella* were enumerated in the cecum. **B)** From the mice in panel A, the lipocalin concentration in feces was measured on days 0, 2, and 4, and in cecum on day 6. **C)** From the mice in panel A, the probiotic *Salmonella* strain ASD203 was enumerated from feces on days 1, 3, and 5, and from the cecum on day 6. The data were transformed and the mean with standard deviation is shown. Statistical significance of two groups being different from each other was determined using an ordinary one-way ANOVA. * = *P* value <0.05, ** = *P* value <0.005, *** = *P* value <0.0005, **** = *P* value <0.00005.

We next performed an experiment in which mice were placed on a high-fat diet and were then infected with either wild-type *Salmonella* or the *fraB* E214A mutant (Fig 7A) F-Asn was gavaged on days zero through three post-infection which caused greater than a 100-fold reduction of the *fraB* mutant. Interestingly, after daily F-Asn gavaging was stopped, the *fraB* mutant CFU recovered to wild-type levels on day 5. Unlike normal mouse chow, the high-fat chow has no F-Asn (detection limit of 50 nM, see Materials and Methods) accounting for the need for F-Asn supplementation to inhibit the *fraB* mutant. When combined with F-Asn, the administration of the *Salmonella* probiotic strain ASD203 had no effect on the wild-type infection but greatly reduced the *fraB* mutant recovered (greater than 10,000-fold). The effect of ASD203 on the *fraB* mutant persisted through day six when the experiment was ended, and indeed, enumeration of ASD203 showed that this strain persisted throughout the experiment (Fig 7C). Most importantly, the combination of F-Asn and the probiotic ASD203 greatly reduced the inflammation caused by the *fraB* mutant (greater than 1000-fold on day 6) (Fig 7B).

Since a genetically modified *Salmonella* strain is less appealing as a probiotic, we tested *E. coli* Nissle, a commercially available probiotic. Like ASD203, *E. coli* Nissle cannot cause inflammation, and is unable to utilize F-Asn, and thus should act as a competitor for nearly all nutrients that *Salmonella* can utilize. *E. coli* Nissle should therefore drive *Salmonella* to utilize a higher proportion of F-Asn. This expectation was confirmed when *E. coli* Nissle, combined with F-Asn, dramatically reduced the number of *Salmonella fraB* E214A mutant bacteria recovered from mice on day 1, but then had progressively less effect on days 3, 5*,* and 6 (Fig 7A). It appears that *E. coli* Nissle did not persist in this experiment, although we did not measure the levels of this probiotic strain since it has no easily selectable antibiotic-resistance marker.

## Discussion

The comprehensive study in this report was inspired by one question: is *Salmonella* FraB a viable drug target? By employing different mouse models on a normal and high-fat chow (± probiotics) and leveraging an integrated workplan encompassing histopathology, mass spectrometry, and molecular biology methods, we provide evidence that FraB is a useful drug target.

The extreme attenuation of *Salmonella fraB*::kan mutants in different mouse models, and the presence of F-Asn in mouse chow and in some human foods, suggests that FraB merits pursuit as an antibacterial target. However, we also discovered that the degree of F-Asn intoxication varies greatly depending upon the *fraB* allele and the mouse model tested (Fig 2 and Fig 3). The *fraB* E214A mutant is our best proxy for a wild-type cell inhibited by a hypothetical FraB inhibitor. In competition experiments, the *fraB* E214A mutant was attenuated 4-fold in strep-treated C57BL/6 mice (at 4 days post-infection), 9-fold in strep-treated Swiss Webster mice (at 4 days post-infection), 8-fold in strep-treated CBA/J mice at day 1 post-infection and no longer detected at 10 days post-infection (Fig 3). In single infections (in strep-treated Swiss Webster mice at four days post-infection), the *fraB* E214A mutant was 48-fold attenuated (Fig 5A). If F-Asn, probiotic (ASD203), or both were provided, the *fraB* E214A mutant was 16-fold, 20-fold, or 630-fold attenuated, respectively; the 630-fold difference is misleading because the wild-type increases in abundance when F-Asn is provided. In these experiments, the *fraB* mutant was recovered at 6.3 x 10^5^, 5 x 10^6^, 1 x 10^6^, and 3.1 x 10^5^ CFU/g (corresponding to 48-, 16-, 20- and 630-fold attenuation). While not as dramatically attenuated as the *fraB80*::kan mutants, the *fraB* E214A mutant is still attenuated. For comparison, the *fraB* E214A mutant is far more attenuated than a SPI1 SPI2 knockout [see the CFU recovered of the *Salmonella* quadruple mutant ASD203 (SPI1 SPI2 Δ*fraR-BDAE ansB*) compared to the CFU recovered of the *fraB* E214A mutant, Fig 5D]. However, a SPI1 SPI2 mutant does not cause inflammation while the *fraB* E214A mutant does. Providing F-Asn to the *fraB* E214A mutant does not reduce the *Salmonella* burden sufficiently to reduce inflammation. However, the co-administration of strain ASD203 further reduces the number of *fraB* E214A mutants in the gut. ASD203 is what we refer to as our ‘probiotic’ strain, a *Salmonella* quadruple mutant (SPI1 SPI2 Δ*fraR-BDAE ansB*). This strain is unable to elicit inflammation due to the mutations in SPI1 and SPI2 but can compete with the *fraB* E214A mutant for all nutrients except F-Asn. Thus, the *fraB* E214A mutant consumes primarily F-Asn and intoxicates itself. The presence of ASD203 reduced the colitis index in the strep-treated Swiss Webster mice infected with the *fraB* E214A mutant from severe to moderate (Fig 5E).

The use of mice that have been pre-treated with streptomycin consistently allows *Salmonella* to colonize and provoke inflammation of the gastrointestinal tract and provide an extremely useful model of *Salmonella*-mediated gastroenteritis [63,64]. However, humans encountering *Salmonella* have not likely been pre-treated with streptomycin. An alternative experimental approach is to use the CBA/J mouse model [34,35]. This strain of mouse, for unknown reasons, allows persistent colonization of the gut by *Salmonella*. Roughly 10% to 30% of the mice become high responders (i.e., high *Salmonella* burden) after six to twelve days and the mice become highly inflamed [6]. These high responders are valuable for studying *Salmonella*-mediated gastroenteritis, but large numbers of mice need to be used inevitably causing large variation in the data. Other groups have recently published that diets high in fat, or high in amino acids, can enhance *Salmonella* colonization and virulence [36,69–71]. Therefore, we directly compared *Salmonella* colonization in the cecum and feces of CBA/J mice on normal chow or high fat diet chow, and Swiss-Webster mice on normal chow but pre-treated with streptomycin (Fig 6). As expected, the variation in *Salmonella* recovered from CBA/J mice on normal chow was quite high, while the numbers recovered from streptomycin-treated Swiss Webster mice were consistent. The high-fat diet did indeed make the number of *Salmonella* recovered from CBA/J mice much more consistent. This change could be a valuable modification of the CBA/J mouse model for future studies.

When we tested the virulence of the *Salmonella fraB* E214A mutant in the CBA/J mice on a high-fat diet, we used single infections in which each mouse only gets one strain of *Salmonella*, either wild-type or mutant (Fig 7). Since the high fat diet lacks F-Asn, we administered 100 μL of 1 M F-Asn each day on days 0–3. We also tested a one-time administration of a ‘probiotic’ strain, either *E. coli* Nissle, or ASD203, on day 0. The *fraB* E214A mutant was recovered in 100-fold lower numbers than wild-type on days 1 and 3, but quickly rebounded on day 5, consistent with the lack of F-Asn administration after day 3. The co-administration of the probiotic strain ASD203 reduced *Salmonella* burden by 100,000-fold and this effect persisted through the end of the experiment at day 6. Inflammation was also greatly reduced, as lipocalin-2 in the feces of mice infected with the *fraB* E214A mutant and the probiotic ASD203 was decreased by >99% compared to the mice infected with wild-type *Salmonella* (day 4 and 6, Fig 7). Other commercially available probiotics may be able to substitute for our *Salmonella* ‘probiotic’ strain in this capacity, and we confirmed this using *E. coli* Nissle, which reduced inflammation by 96% on day 4 (Fig 7). The probiotics were only administered on day 0 and while ASD203 persisted through day 6, the effects of *E. coli* Nissle did not persist. It is possible that daily administration of *E. coli* Nissle would solve this problem.

Resistance to F-Asn arises among *fraB* mutants during mouse infection, in the form of *fraA* and *fraD* mutations. These arise quickly enough to make FraB inhibitors a poor choice for widespread agricultural use. However, the results from mouse models in this report indicate that *Salmonella* burden and inflammation can be greatly reduced despite the appearance of resistant mutants during the infection. This reduction in *Salmonella* burden would provide time for the adaptive immune system to clear the infection and for the normal microbiota to recover and restore colonization resistance. Thus, for human use, or very limited agricultural use, it appears that FraB may be an effective therapeutic target, especially when combined with a probiotic strain that can compete with *Salmonella* for nutrients other than F-Asn.
